# Toward the Development of a Pan-Lyssavirus Vaccine

**DOI:** 10.3390/v16071107

**Published:** 2024-07-10

**Authors:** Sabrine Ben Hamed, Jacob F. Myers, Anisha Chandwani, Christoph Wirblich, Drishya Kurup, Nir Paran, Matthias J. Schnell

**Affiliations:** Department of Microbiology and Immunology, Sidney Kimmel Medical College, Thomas Jefferson University, Philadelphia, PA 19107, USAnir.paran@jefferson.edu (N.P.)

**Keywords:** rabies virus, lyssavirus, Mokola virus, Irkut virus, epitope-based vaccine, pan-lyssavirus vaccine

## Abstract

In addition to the rabies virus (RABV), 16 more lyssavirus species have been identified worldwide, causing a disease similar to RABV. Non-rabies-related human deaths have been described, but the number of cases is unknown, and the potential of such lyssaviruses causing human disease is unpredictable. The current rabies vaccine does not protect against divergent lyssaviruses such as Mokola virus (MOKV) or Lagos bat virus (LBV). Thus, a more broad pan-lyssavirus vaccine is needed. Here, we evaluate a novel lyssavirus vaccine with an attenuated RABV vector harboring a chimeric RABV glycoprotein (G) in which the antigenic site I of MOKV replaces the authentic site of rabies virus (RABVG-cAS1). The recombinant vaccine was utilized to immunize mice and analyze the immune response compared to homologous vaccines. Our findings indicate that the vaccine RABVG-cAS1 was immunogenic and induced high antibody titers against both RABVG and MOKVG. Challenge studies with different lyssaviruses showed that replacing a single antigenic site of RABV G with the corresponding site of MOKV G provides a significant improvement over the homologous RABV vaccine and protects against RABV, Irkut virus (IRKV), and MOKV. This strategy of epitope chimerization paves the way towards a pan-lyssavirus vaccine to safely combat the diseases caused by these viruses.

## 1. Introduction

Lyssaviruses belong to the *Lyssavirus* genus within the Rhabdoviridae family. In addition to rabies virus (RABV), 16 additional viruses from the lyssavirus genus have been identified in Africa, Asia, Australia, and Europe. These viruses are genetically divergent, have distinct antigenic properties, and are divided into three phylogroups: phylogroup I, phylogroup II, and an unclassified group [1,2,3]. Despite their genetic and antigenic differences, all lyssaviruses cause lethal disease in humans with similar symptoms to rabies.

Dogs remain the leading cause of human exposure to classical RABV, especially in areas where canine vaccination programs are not effectively enforced, such as in Asia and Africa. Based on the World Health Organization (WHO) estimates, about 59,000 people die from rabies each year, and 40% of fatalities are children under the age of 15 [4].

Intensive efforts in North America to eradicate rabies were successful, resulting in fewer annual human deaths [2]. However, classical rabies persists in wild animals like raccoons (Procyon lotor), skunks (Mephitis), jackals (Canis), foxes (Vulpes), and bats (Chiroptera), and new lineages of RABV were discovered in South America, expanding the list of reservoirs. The CDC reported recently that out of 10 rabies cases, 7 died from bat-mediated rabies from 2009 to 2018 [5].

Recently, more lyssavirus species have been identified to cause fatal encephalitis in humans, such as Australian bat lyssavirus in 2000 [6,7], Duvenhage virus in South Africa in 2006 [8], IRKV in Russia in 2019 [9], European bat 1 Lyssavirus in 2022 [10], and Mokola virus and Lagos bat viruses in South Africa respectively in 2014 and 2006 [11,12,13]. Following exposure to a lyssavirus, the virus infects the tissue at the exposure site followed by retrograde transport through the peripheral nervous system, eventually reaching the central nervous system (CNS) and causing encephalomyelitis. In humans, the disease typically begins with non-specific symptoms (fever, malaise, and headache), followed by anxiety and agitation. When the virus reaches the CNS, it spreads outward in the peripheral nervous system in a phenomenon called centrifugal spread, using the nerves to reach tissues like the salivary glands, and proceeds until the entire nervous system fails, causing paralysis and rapid death [14].

The currently licensed rabies vaccinations are effective against classical RABV when given properly. For pre-exposure, the vaccine is given on day 0,7 and 21 or 28, while the post-exposure schedule varies between a five- or four-dose schedule [15,16]. The correlate of protection for rabies vaccines is determined by the level of neutralizing antibodies, which target the viral surface glycoprotein (RABV G) [17].

Being the only target of neutralizing antibodies, the unique surface RABV G of rabies virus has been the subject of studies determining its crystal structure before and after fusion and identifying its antigenic sites: I II, III, IV, and a minor antigenic site “a” [18,19,20]. Antigenic site 1 (AS1) is a linear epitope, which was characterized using a monoclonal antibody (mAb) CR57 to bind six residues KLCGVL 226–231 of RABVG [20,21]. The other antigenic sites are conformational and non-continuous. Lyssavirus glycoproteins share 57–78% similarity [19,22], impacting the cross-protection against non-rabies lyssaviruses after RABV vaccination. Previous studies have shown that the rabies vaccine protects against RABV with a neutralizing titer above 0.5 international unit [IU] and cross-protects against other phylogroup I lyssaviruses with different neutralizing antibody titer cut-offs [20]. However, the current RABV vaccines do not induce neutralizing antibodies or protect against lyssaviruses from phylogroup II, such as MOKV or the very divergent lyssaviruses assigned to the unclassified phylogroup [23,24,25].

This limitation of the current rabies vaccine, as well as the ongoing emergence of non-rabies lyssaviruses [3,26], underlines the unassessed dangers and the need for a more broadly effective lyssavirus vaccine able to prevent human rabies diseases caused by viruses of the lyssaviruses genus. Previously, our lab designed a pan-lyssavirus vaccine where domains between RABV G and MOKV G were exchanged, creating a chimeric RABV G, but vaccination with that chimeric G-containing vaccine compromised protection for other phylogroup I lyssaviruses, leading us to try to redesign the vaccine in a way that better preserved phylogroup I protection [1]. Using recent developments in structural biology and the solved structures of RABVG and MOKVG [18,19,27], our paper presents the development of a novel RABV-G-based vaccine harboring a chimeric AS1, “ RABVG-cAS1”, in which a single AS1 from MOKVG has been introduced into RABVG, replacing the authentic AS1.

## 2. Materials and Methods

### 2.1. Animals and Care

This study was carried out in strict adherence to the recommendations of the Guide for the Care and Use of Laboratory Animals and the guidelines of the National Institutes of Health, the Office of Animal Welfare, and the United States Department of Agriculture. All animal work was approved by the Institutional Animal Care and Use Committee (IACUC) at Thomas Jefferson University (protocol ID 01940-1). All procedures were performed under isoflurane anesthesia by trained personnel under the supervision of veterinary staff. Swiss Webster mice (Charles River) were housed in cages in groups of five under controlled conditions of humidity, temperature, and light (12 h light/12 h dark cycles). Food and water were available ad libitum.

### 2.2. Cells

HEK 293T Human Embryonic Kidney 293, BSR a clone of Baby kidney Hamster cells, both available from our laboratory, and Beas.2B (lung cells, ATCC^®^ CRL-9609™, Manassas, VA, USA) were cultured using DMEM (Gibco, Grand Island, NY, USA) with 5% Fetal Bovine Serum (FBS) (Atlanta-Biologicals^®^, Minneapolis, MN, USA) and 1% penicillin–streptomycin (P/S) (Gibco, Grand Island, NY, USA). Mouse neuroblastoma (NA) cells (available from our laboratory) were cultured in RPMI (Corning^®^) with 5% FBS and 1× P/S. Cell cultures were maintained at 37 °C with 5% CO_2_, whereas virus-infected cells were cultured at 34 °C with 5% CO_2_.

### 2.3. Antibodies

Human anti-RABVG 4C12 mAbs (provided by Dr. Scott Dessain, Lankenau Institute for Medical Research, Wynnewood, PA, USA), mouse anti-MOKVG mAbs 3A5 (made by OCMS-bio, King of Prussia, PA, USA, and Schnell lab, Philadelphia, PA, USA), mouse anti-MOKV-G polyclonal antibody, and rabbit anti-ribonucleoprotein (RNP) polyclonal antibodies were used in this study. We also used goat anti-mouse IgG Fc-HRP (Southern Biotech, Birmingham, AL, USA), goat anti-human IgG Fc-HRP (Southern Biotech), and fluorescein isothiocyanate (FITC)-conjugated anti-rabies virus nucleoprotein (RABV N).

### 2.4. Viruses

In this study, we used wildtype (wt) Mokola virus (MOKV, isolate 252/97) (GenBank: GQ500112.1); wt IRKV both provided by Dr. Todd Smith, US Centers for Disease Control and Prevention, Atlanta, GA, USA (GenBank: EF614260.1); and the RABV CVS-N2C strain (GenBank: HM535790.1). All work with wtMOKV and wt IRKV was performed under BSL3 conditions at Thomas Jefferson University. Work on RABV-N2C- and RABV-based vaccine strains was conducted under BSL2/3 conditions.

A recombinant Vesicular Somatic Virus (VSV) (Indiana strain) and a recombinant VSV∆G expressing MOKVG and RABVG were used. The use of the above-mentioned viruses was approved by the institutional biosafety committee at Thomas Jefferson University (IBC protocol ID: 22-09-560).

### 2.5. In Silico Design

**Figure 1:**
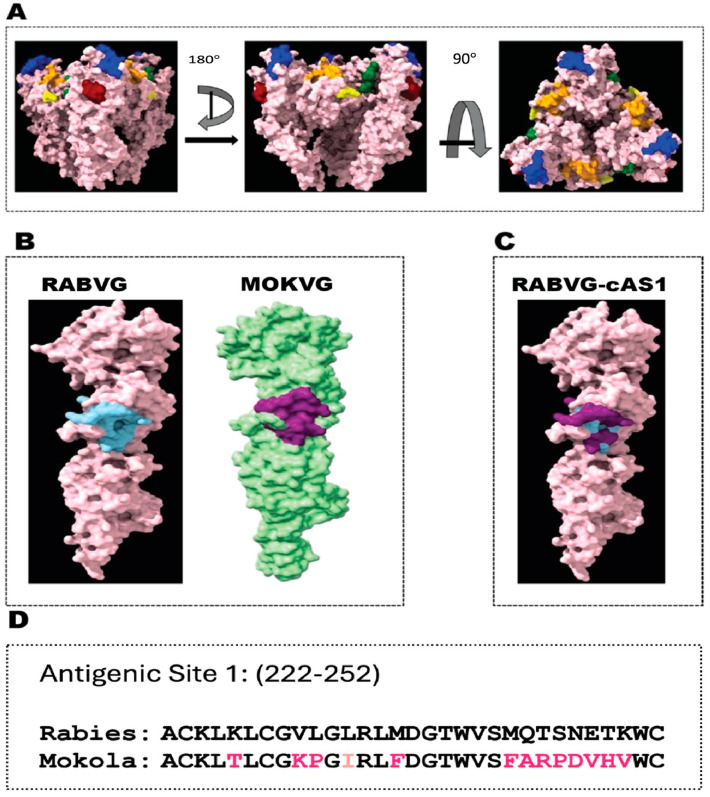
The in silico study and modeling of the epitope–based pan–lyssavirus vaccine. (**A**) The structure of the RABVG trimer in the pre-fusion state showing the different antigenic sites as previously described [15]. The antigenic site 1: 226–231 is highlighted in maroon, antigenic site 2: 34–42, 198–200 is highlighted in blue, antigenic site 3: 330–338 is highlighted in orange, antigenic site 4: 261–264 is highlighted in green, and minor antigenic site a: 342–343 highlighted in yellow. (**B**) The antigenic site from amino acid 222–252 presented in bright blue in RABV-G and purple in MOKV-G. (**C**) The structure of RABVG-cAS1, with colors representing the contribution of MOKV-G (purple) to the combined chimeric G. (**D**) The amino acid alignment of the antigenic site (222–252) between RABVG and MOKVG. Conservative mutations are colored with bright pink and, non-conservative mutations with light pink.

The in silico studies were performed using Chimera X software (version: 1.6rc202304140213). Initial structural comparisons were made between the glycoprotein structures stemming from Yang et al. and Belot et al. [18,27] shown in Figure 1. RABV-G post-fusion structure (PDB ID: 6LGW) [1] and MOKV-G post-fusion structure (PDB ID: 6TMR) [27] were compared and found to be very similar. The established antigenic sites in RABV-G were compared between RABV-G pre-fusion and post-fusion structures (PDB IDs: 6LGX and 6LGW, respectively) to establish which antigenic sites appeared to be involved in the fusion machinery.

#### 2.5.1. cDNA Construction of Vaccine Vectors

The vaccine vector RABV has been described previously [28]. The vaccine vector BNSP∆G-MOKVG was cloned by inserting the codon-optimized (co) MOKV-G gene between the nucleoprotein (N) and the phosphoprotein (P) of the RABV vector with its G gene deleted (RABV∆G) as previously described [29]. A recombinant RABV-G DNA was designed to encode the RABV-G gene in which the sequence encoding the antigenic site from amino acids 222–252 was replaced by the corresponding sequence of MOKVG. The recombinant chimeric rabies glycoprotein DNA was synthesized by Genescript. A polymerase chain reaction (PCR) fragment encoding for RABVG-cAS1 was amplified using primers SB.38 and SB.39 (Table 1). The fragment was inserted between the BsiWI and Nhel sites of RABV using In-Fusion cloning (Takara Bio Inc., Mountain View, CA, USA). The recombinant sequence was confirmed by Sanger sequencing.

#### 2.5.2. Recovery of Recombinant Vectors

To recover the recombinant rabies viruses, 70% confluent BSR cells were transfected with the full-length viral cDNA clones (RABVG-cAS1, RABV∆G-MOKVG), along with plasmids encoding the RABV N, P, and L genes and a plasmid expressing T7 RNA polymerase in a master mix with X-tremeGENE 9 transfection reagent (Roche Diagnostics, Indianapolis, IN, USA ) and Opti-MEM medium as previously described [30]. Successful recovery was determined using rabies virus focus-forming assay. Briefly, four days post-transfection, the supernatant from each transfected well of the 6-well plate was transferred into BSR cells in a 12-well plate. Two days later, cells in the 12-well plate were fixed with 80% acetone and stained with a FITC-conjugated antibody against RABV N (Fujirebio Diagnostics, Inc, Malvern, PA, USA). Fluorescent microscopy and a cell imager multimode reader BioTek Cytation 5 (Aligent, Santa Carla, CA, USA) were used to visualize the appearance of viral foci, indicating the successful recovery of the recombinant RABVG-cAS1. A further confirmation of the recombinant glycoprotein‘s sequence was determined by RNA extraction using PureLink RNA Mini Kit, Invitrogen from the supernatant, RT-PCR using SuperScript™ IV One-Step RT-PCR (Invitrogen, Waltham, MA, USA) using the primers (RP951 and RP952), and Sanger sequencing using the same primers.

#### 2.5.3. Sucrose Purification and Inactivation of Virus Particles

RABVG-cAS1, RABV, and RABV∆G-MOKVG were grown on a large scale by infecting BSR cells in Corning^®^ 3313 CellSTACK^®^ at an MOI of 0.001. The supernatant of each virus was collected every four days for a total of 5 harvests. The titers of the harvests were determined using the rabies virus focus-forming assay, and the harvests with the highest titers were pooled for each virus and concentrated with an Amicon^®^ 300 mL stirred cell concentrator (MilliporeSigma^®^, Burlington, MA, USA) using a 500 kDa exclusion Polyethersulfone (PES) membrane (MilliporeSigma^®^, Burlington, MA, USA). Concentrated supernatants were then centrifuged for 2 h at 25,000 rpm through a 20% sucrose cushion using an SW32 Ti rotor (Beckman, Sharon Hill, PA, USA). The virion pellets were resuspended in phosphate-buffered saline (PBS), and protein concentrations were determined using a bicinchoninic acid (BCA) assay kit (Pierce, Waltham, MA, USA). The virus particles were chemically inactivated with β-propiolactone (BPL) at a dilution of 1:2000 overnight at 4 °C. The BPL in the virus preparation was inactivated the next day by hydrolysis at 37 °C for 30 min. The absence of infectious particles was verified by inoculating BSR cells in a T25 vessel with 10 μg of BPL-inactivated virus for 3 passages. Inoculated cells were fixed and stained with FITC-conjugated anti-RABV N mAb and visualized by fluorescence microscopy for the presence infection’s foci.

#### 2.5.4. Western Blot Characterization

Sucrose-purified virus particles (1 µg each) were denatured by boiling at 95 °C for 10 minug of the TLR-4 agonist synthetic Monop in a final concentration of 1× of Laemmli sample buffer supplemented with 2% of β-mercaptoethanol (BME). Lysates were resolved on 10% SDS-PAGE. The gel was transferred onto a nitrocellulose membrane in a transfer buffer (192 mM glycine, 25 mM Tris, 20% methanol) for Western blot mini-analysis. Blots were then blocked for 1 h at room temperature in 5% nonfat dry milk dissolved in PBS-T (0.05% Tween^®^ 20 [MilliporeSigma^®^]). After three washes, membranes were probed with primary antibody in a solution of 5% bovine serum albumin (BSA) in PBS. Human anti-RABVG mAbs at a dilution of 2 µg/mL, mouse anti-MOKVG polyclonal sera at a dilution of 1:5000, and rabbit anti-RABV-RNP polyclonal antibodies (1:5000) were used as primary antibodies and incubated overnight. The next day, the blots were washed with PBS-T before being incubated at 1:20,000 with horseradish peroxidase (HRP)-conjugated goat anti-mouse (Goat anti-mouse IgG Fc HRP Southern Biotech) and goat anti-human IgG (SouthernBiotech, 2040-05). Proteins were detected using the SuperSignal West Dura Chemiluminescent substrate (Pierce^®^) and imaged using the FluorChem R system (proteinsimple^®^, San Jose, CA, USA).

#### 2.5.5. Production, Titration, and Sequencing of Challenge Viruses

Wt MOKV, wt IRKV, and RABV CVS-N2C were grown in 70% confluent NA cells cultured in RPMI supplemented with 5% FBS and 1% penicillin–streptomycin at an MOI of 0.001. Lyssaviruses were collected at three different times post-infection (day 5, day 8, and day 11).

For titration, each virus was tittered separately in triplicate. Briefly, 10-fold serial dilution was performed in round-bottom 96-well plates. Viruses were added on a monolayer of NA cells (40,000 cells/well), cultured in RPMI supplemented with 5% of FBS and 1% of P/S, and incubated at 34 °C. Two days later, plates were fixed with 80% cold acetone overnight, dried, and stained with the lyssavirus cross-reactive FITC-conjugated antibody against RABV N (Fujirebio Diagnostics, Inc.). Titers were determined by counting the foci-forming unit (ffu/mL) using a fluorescence microscope. Each lyssavirus virus stock was validated by RT-PCR and the sequencing of their respective glycoproteins using the following primers: SB.28, SB.29, SB.30, SB.31, SB.42, and SB.43 (Table 1).

#### 2.5.6. Immunization and Challenge Experiments

Groups of five 8–10-week-old female Swiss Webster mice bought from Charles River Laboratories were immunized intramuscularly (i.m) with 10 µg of the following BPL-inactivated rabies vaccines: RABV-cAS1, BNSP∆G-MOKVG, and RABV. Each vaccine was adjuvanted with 5 µg 3D (6 A)-PHAD and 2% SE in a final volume of 100 µL of PBS per mouse, 50 µL per hind limb. Control mice received an intramuscular injection of PBS. The intramuscular challenge consisted of injecting 100 µL (50 µL each hind leg). Intranasal challenge was performed by instilling 20 µL into both nostrils. Both challenges were conducted under 3% isoflurane/dioxygen gas anesthesia.

Mice were primed on day 0 and boosted on day 28. To assess vaccine immunogenicity and conduct further testing, sera were obtained by retro-orbital bleeds under isoflurane anesthesia on the following days: Day of the Prime (D0), day of the booster (D28), a week after the boost (day 35), and a week before the challenge (day 91). Three months after the prime immunization, mice were challenged with lethal doses of each of the following lyssavirus: 10^4^ ffu of wt MOKV intranasally, 10^5^ ffu of wt IRKV intramuscularly, and 5.5 10^6^ ffu of rabies virus CVS-N2C intramuscularly. Following infection, mice were monitored twice a day for weight and signs of disease such as ruffled fur, lethargy, hind limb paralysis, and seizures. Mice who lost more than 20% of their initial weight, developed paralysis, or had seizures were deemed to have reached the humane endpoint and were euthanized. After 28 days from the day of challenge, all mice were euthanized via heart puncture, and their brains were collected.

### 2.6. Production of Soluble RABVG- and MOKV-G

To generate RABV-G and MOKV-G soluble antigens, Beas.2b cells were infected with rVSVΔG-GFP-RABVG and rVSV∆G-GFP-MOKVG, respectively, in OptiPRO™ SFM (Gibco, Grand Island, NY, USA) at an MOI of 0.01. Three days post-infection, virus supernatants were filtered through a 0.45µm filter, concentrated using an Amicon^®^ Stirred Cells concentrator, and ultracentrifuged through a 20% sucrose cushion at 25,000 rpm for two hours (Beckman). Pellets were re-suspended in PBS with 2% OGP (Octyl β-D-glucopyranoside) detergent and shaken at room temperature for 30 min. The mixture was then centrifuged at 3000× *g* for 10 min, and the supernatant was collected. To separate the soluble antigens from the rVSV particles, ultracentrifugation was performed in a Beckman SW 55 Ti rotor at 45,000 rpm for 1.5 h. The supernatant containing soluble RABVG and MOKVG was evaluated for protein concentration by BCA (Pierce) and purity by Sypro Ruby stain and Western blot against RABV-G and MOKVG.

#### 2.6.1. Enzyme-Linked Immunosorbent Assay (ELISA)

To test for anti-RABVG and anti-MOKVG humoral responses, the titer of anti-RABV-G and MOKV-G IgG antibodies was determined by ELISA with mice sera from days 0, 28, 35, and 91 post-vaccination. Individual serum samples were analyzed in triplicate, except mock group sera, which were pooled.

Soluble RABVG and MOKVG antigens were diluted in coating buffer (50 mM Na_2_CO_3_ [pH 9.6]) at 500 ng/mL for RABVG and 750 ng/mL for MOKVG and used to coat immulon 4HBX plates (USA scientific Inc, Ocala, FL, USA) 96-well plates and incubated overnight at 4 °C. The next day, plates were washed three times with PBST (0.05% Tween 20 in 1× PBS) and incubated for 2 h at room temperature with a blocking buffer (5% nonfat dry milk powder in 1× PBST). The plates were washed three times with PBST before being incubated overnight at 4 °C with 3-fold serial dilutions of sera from immunized mice beginning with a starting dilution of either 1:50, 1:450, or 1:1350 to reach the endpoint titer. Sera were diluted in a buffer containing 0.5% BSA and 0.05% NaN3 in PBST. The next day, plates were washed, and 100 µL of secondary antibody (horseradish peroxidase-conjugated goat anti-mouse IgG-Fc [Jackson ImmunoResearch, Cat# 115-005-008 West Grove, PA, USA]) was diluted in PBST to 25 ng/mL then added to the plates. Two hours later, plates were washed and developed using 200 µL/well of o-Phenylenediamine Dihydrochloride substrate (ThermoFisher, Norristown, PA, USA) for 15 min. The reaction was ended using 0.6 M H_2_SO_4_ per well. The optical density (OD) was measured at 490 nm (experimental) and 630 nm (background) using a BioTek ELx800 plate reader (BioTek, Winooski, VT, USA) with Gen5 software (Version 3.10) to calculate the delta values between the experimental and background readings. ELISA results were analyzed using GraphPad Prism 9 software to determine the EC50 values of antibodies in mouse sera.

#### 2.6.2. Rabies Virus Neutralization Assay: Rapid Fluorescent Foci Inhibition Test RFFIT

To investigate the presence of rabies-neutralizing antibodies, the RFFIT was performed as previously described [31]. Briefly, flat-bottom 96-well plates were seeded with BSR cells at a cell density of 30,000 cells per well in DMEM containing 5% FBS and 1% penicillin–streptomycin. Sera collected on day 91 were heat-inactivated at 56 °C for 30 min before being serially diluted 3-fold (in triplicate) in opti-MEM (Gibco) with a starting dilution range of 1:50 to 1:12,150. In addition, the WHO standard rabies immune globulin was diluted 3-fold, beginning at 2 international units per milliliter. A predetermined volume of RABV challenge strain CVS-11, to achieve a 90% infection of confluent BSR cells, was added to the sera plates followed by incubation for one hour at 34 °C. The total volume of the virus/sera mixture was added to BSR cells, which were subsequently incubated for 22 h. To determine the percentage of neutralization, plates were fixed with 80% acetone and stained with FITC anti-RABV-N antibody. The percentage of infection was determined by fluorescence microscopy and by a Cytation5 reader (Agilent BioTek, Santa Carla, CA, USA). The 50% endpoint titers were calculated using the Reed–Muench method and converted to IU/mL by comparing them to the WHO standard.

### 2.7. Non-Rabies Lyssavirus Neutralization Assay

Neutralization titers against wt MOKV and wt IRKV viruses were determined similarly to that of rabies virus with the following changes. The assays were performed under biosafety level 3 (BL3) conditions. Neuroplasma NA cells were used at a density of 45,000 cells/well. Plates were fixed for 22 h post-infection with cold 80% acetone overnight, dried, and stained with 80uL of FITC-conjugated anti-RABVN antibody. The determination of 50% neutralizing titers (NT50) was similarly evaluated using a manual inverted microscope and a Cytation5 reader (Agilent BioTek).

### 2.8. RNA Extraction and Reverse Transcription Polymerase Chain Reaction (RT-PCR)

Brain tissues were collected from challenged mice in Omni tubes containing 1.4 mm ceramic beads (Omni International, Cat#: 19-627D, Kennesaw, GA, USA) and 600 uL of DMEM, which were subsequently homogenized twice for 90 s each until all the brains were completely dissolved. A total of 250 uL of homogenized brain tissue was mixed with 750 uL of TRIzolReagent and incubated overnight at 4 °C. The next day, RNA extraction was performed using Trizol reagent and PureLink RNA Kit (Invitrogen, Waltham, MA, USA). For RNA extraction from the virus suspension, the same process was carried on as described above, without the homogenization step and with TriZolLS. The RNA concentration was quantified using NanoDrop (Thermofisher, Norristown, PA, USA). The presence of RNA encoding the lyssavirus glycoproteins (RABVG, MOKVG, and IRKVG) in the brain was detected by one-step RT-PCR. Total RNA (100 ng) was amplified using the SuperScript™ III One-Step RT-PCR System with Platinum™ Taq DNA Polymerase according to the manufacturer’s instructions and primers listed above in the virus production section. The RT phase consisted of a 10 min incubation period at 55 °C, followed by a 2 min inactivation period at 98 °C. The PCR cycling conditions included an initial denaturation of 98 °C for 10 s followed by 25 cycles of 55 °C for 10 s and 72 °C for 1 min, with a final extension step at 72 °C for 5 min. RT-PCR reactions were performed in a Labnet MultiGene™ OptiMax thermal cycler, and the reactions were subsequently analyzed by DNA gel electrophoresis.

### 2.9. Statistical Analysis

GraphPad Prism 9 was used for all statistical analyses on log-transformed data. In Elisa EC50 titers, RFFIT, and neuralization, an ordinary one-way ANOVA was used with a post hoc analysis applying the Tukey Multiple comparison test. The log-rank Mantel–Cox test was performed to compare the survival of different groups in each virus challenge.

## 3. Results

### 3.1. The In Silico Design of the Epitope-Based Rabies Vaccine

In this study, we aimed to broaden the immunogenicity of a current RABV vaccine towards distant lyssaviruses. To fulfill this goal, the distribution of the antigenic sites as defined by Yang et al. [18] were visualized and primarily found on the apical head regions of the G protein (Figure 1A). For structural reasons, AS1 was chosen as the candidate for designing our vaccine. The structure of RABVG-cAS1 where the AS1 of MOKVG was introduced in RABVG is illustrated in Figure 1B,C.

### 3.2. Generation of Recombinant Rabies Vector Expressing Chimeric Glycoprotein with Mokola Antigenic Site 1 (RABV-cAS1)

**Figure 2:**
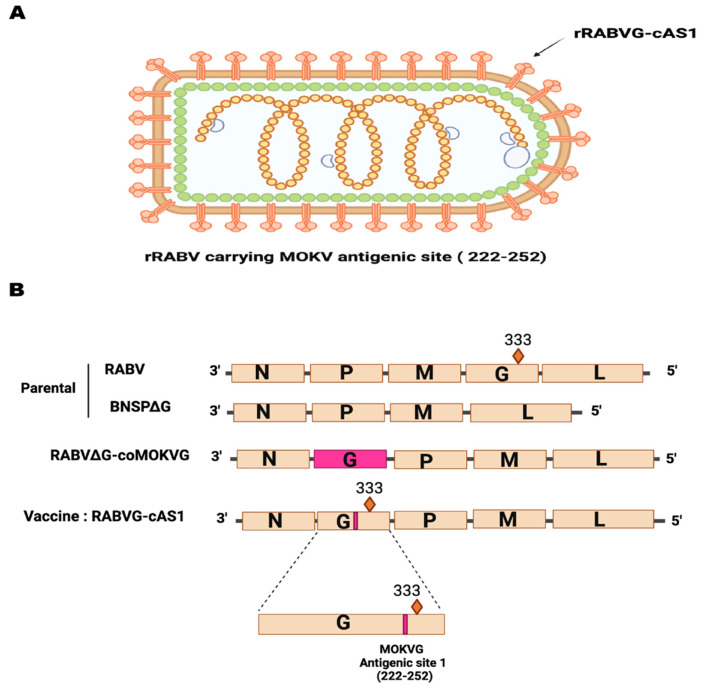
rRABVG–cAS1 genome and rabies virus-based vaccines and vector maps. (**A**) Schematics of the genomic structure of the rRABVG-cAS1 virion: RBVG-cAS1 glycoprotein (orange), the lipid bilayer (brown), matrix protein (green), Nucleoprotein (yellow), polymerase (big grey circle), phosphoproteins (small grey crescents). (**B**) Recombinant rabies virus-based vaccines and their vector controls RABV and BNSP∆G. The marked 333 in RABV is the R333E attenuation. Recombinant rabies glycoprotein carrying MOKVG antigenic site (222–252) was inserted between N and P. Figure created using Biorender.com (accessed on 2 May 2024).

To construct a recombinant RABV expressing RABVG with the AS1 from MOKVG (Figure 2A), we used the RABV vector BNSP333. BNSP333 is an established vector that was derived from the RABV vaccine strain SAD B19 and further attenuated through an arginine-to-glutamate mutation at amino acid 333, resulting in reduced neurotropism [32,33,34,35]. The gene encoding the chimeric glycoprotein AS1 contains MOKVG’s AS1 from amino acid 222 to amino acid 252. The alignment of RABVG and RABV-expressing Mokola AS1 shows the presence of both conservative and non-conservative mutation (Figure 1D). As a control vector, we also constructed a BNSP∆G-MOKVG where the RABVG was replaced with a codon-optimized MOKVG between the N and P genes (Figure 2B) [29].

### 3.3. RABV-cAS1 Is Recovered and Successfully Incorporated

**Figure 3:**
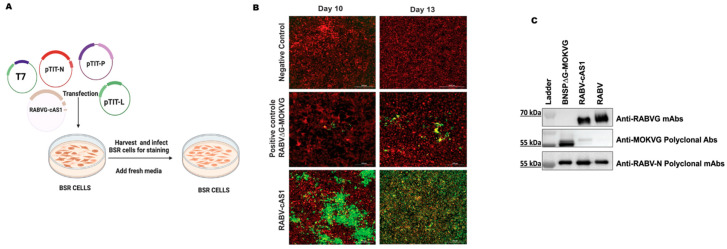
Recovery of RABV-cAS1 and virus characterization. (**A**) Transfection of rabies plasmids with RABVG-cAS1 construct (brown color) with T7 promoter in BSR cells. Figure created using Biorender.com. (**B**) Cell staining with RABV-N-FITC antibody (infected cells with RABV-cAS1 are in green). Images were taken from BioTek Cytation 5 Cell Imaging Multimode Reader. (**C**) Western blot of rabies sucrose-purified virions (controls: RABV, recombinant RABVG-MOKVG, and the recombinant RABVG-cAS1 using anti-human RABVG (4C12), anti-mouse MOKVG polyclonal antibodies, anti-rabbit RABV-N polyclonal antibodies.

To recover the recombinant virus RABVG-cAS1, BSR cells were transfected with cRABV-cAS1 and support plasmids encoding the RABV N, P, and polymerase L genes controlled by the T7 promoter, as previously described (Figure 3A) [30]. Ten days post-transfection with cRABVG-cAS1, we observed RABV-N positively stained foci of RABV infected cells that spread throughout the culture by day 13, indicating the successful recovery of the recombinant virus RABVG-cAS1. To confirm the incorporation of the G proteins into the viral particles, a Western blot analysis of the sucrose-purified virions RABV, RABV-cAS1, and RABV∆G-MOKVG was performed. The incorporation of the chimeric antigen into the RABV particles was detected using specific antibodies against RABV and MOKV (anti-human RABVG-mAb [4C12] and anti-mouse MOKVG polyclonal antibodies). Probing with an RNP-specific antibody demonstrated similar levels of N for all viruses (Figure 3).

### 3.4. Inactivated RABV-cAS1 Vaccine Induces Humoral Response in Mice against RBVG and MOKVG

**Figure 4:**
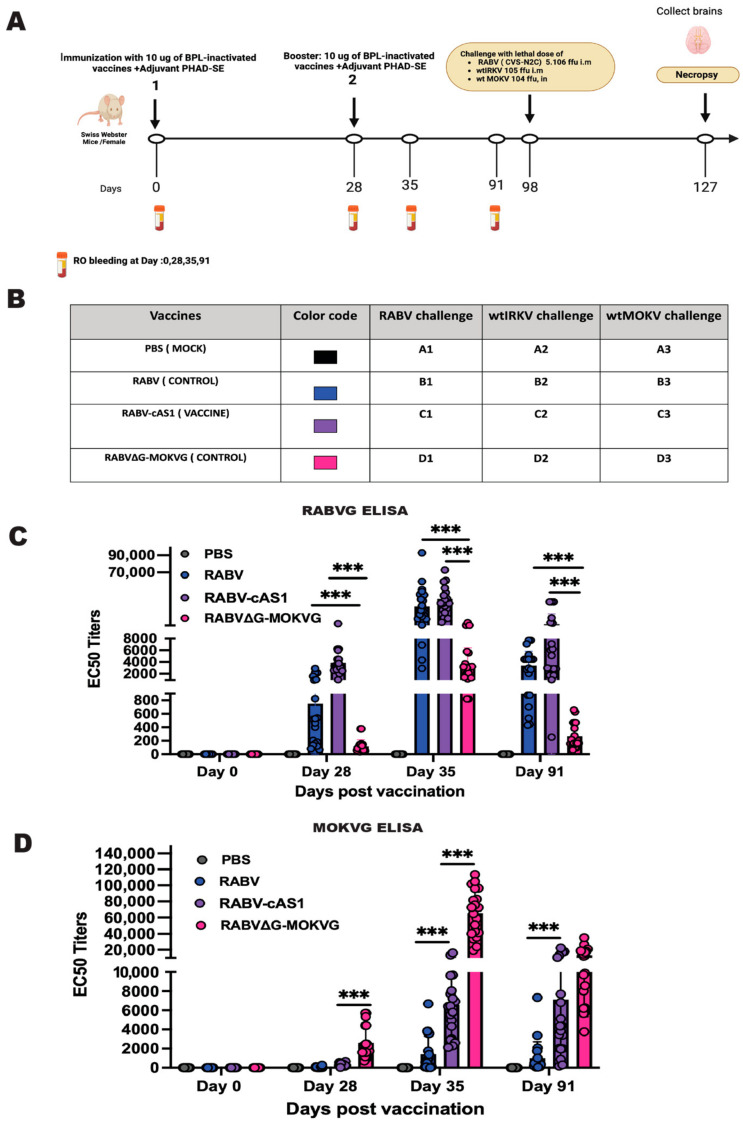
The RABV-cAS1 vaccine elicits humoral responses against RABVG and MOKVG. (**A**) The study design for the RABV-cAS1 vaccine protection study against lethal doses of lyssaviruses from phylogroups I and II. The schematic shows the immunization and blood draw schedule. Groups of 5 female Swiss Webster mice were immunized with 10 µg/dose of BPL-inactivated vaccines adjuvanted with 5 µg of PHAD in 2% SE per dose. In the figure, 1 represents the first immunization, 2 the boost, blood vials indicate the bleeding schedule, and the brain denotes the necropsy and brain harvest at the end of the study. (**B**) A table showing the vaccine groups used in this study, along with their color codes and lyssavirus challenges. Created using Biorender.com. (**C**,**D**) EC50 Elisa titers over time for the RABVG (**C**) and MOKVG (**D**) antigens. Error bars represent the mean with SD for the mice groups. Elisa was carried out in triplicate for each sample. Statistical differences between groups were analyzed using an ordinary one-way ANOVA with Tukey’s Multiple comparison test. A 3-star significance level indicates a *p*-value below 0.0002 (*** *p* < 0.0002).

To investigate the immunogenicity of the RABV-cAS1 vaccine against the RABVG and MOKVG antigens, groups of five Swiss Webster mice were vaccinated intramuscularly (i.m.) with 10ug of BPL-inactivated vaccine adjuvanted with 5 ug of the TLR-4 agonist synthetic Monophosphoryl Lipid A (MPLA) 3D(6A)-PHAD in a 2% squalene-in-oil emulsion (SE) (Figure 4A). The mice were bled on days 0, 28, 35, and 91. Sera were tested using an enzyme-linked immunosorbent assay (ELISA) for RABV G and MOKV G. To prevent cross-reactivity with other RABV or MOKV proteins contaminating the stripped G proteins, soluble glycoproteins were purified from a recombinant VSV particle that contained RABVG or MOKVG. Antibody titers to RABVG were detected at similar levels for sera from mice immunized with RABV and RABVG-cAS1 after prime vaccination (day 28) and were about 10-fold increased after the boost (day 35). In contrast, vaccination with RABV∆G-MOKVG induced an almost undetectable immune response to RABV-G that was still 10-fold lower after the boost compared to the other two vaccines (day 35) (Figure 4C). In addition to the strong immune response detected after RABVG-cAS1 vaccination, the chimeric vaccine additionally induced a robust MOKV G antibody response, which was not detected for the RABV vaccine. Meanwhile, the anti-MOKVG EC50 titers of RABVG-cAS1-vaccinated animals were significantly lower than that of RABV∆G-MOKVG-vaccinated controls; on day 91, both groups elicited about 10,000 EC_50_ titers to MOKVG with no significant difference (Figure 4D).

### 3.5. Rabvg-cAS1 Vaccine Induces Neutralizing Antibodies against Phylogroups I and II Lyssaviruses

**Figure 5:**
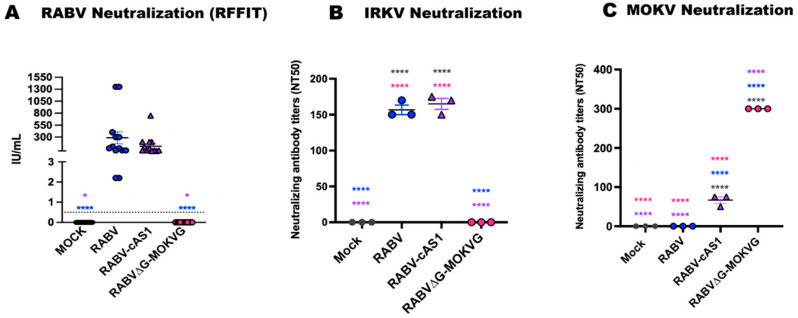
RABV-cAS1 vaccine elicits neutralizing antibodies against RABV, IRKV, and MOKV. (**A**) Rapid fluorescent focus inhibition test (RFFIT) using individual sera from mice immunized with different rabies-based vaccines against RABV strain CVS-11. Dotted line represents 0.5 IU/mL, WHO’s recommended protective threshold. Figure shows 50% neutralization titers (NT50) of rabies-based vaccines against wt IRKV (**B**) and wt MOKV (**C**) using pooled sera from 3 different immunized groups for each vaccine. Error bars represent standard deviation (SD). Statistical differences between groups were determined using ordinary one-way ANOVA with Tukey’s Multiple comparison test with (**** *p* < 0.0001, * *p* < 0.0332).

The results presented above indicate that the vaccine induces strong immune responses against both RAB G and MOKV G. To further elaborate upon the protective potential of the chimeric vaccine, we evaluated the sera in virus neutralization assays (VNAs) with a panel of different lyssaviruses. To that aim, serum samples collected on day 91 from mice immunized with the three vaccines were evaluated for the ability to neutralize RABV strain CVS-11 using the rapid fluorescent focus inhibition test (RFFIT) (Figure 5A). Both RABV-cAS1- and RABV-vaccinated mice induced similar RABV-neutralizing antibody titers that were well above the threshold of 0.5 international units per milliliter (IU/mL), considered protective by the WHO [36] (Figure 5A).

We further determined the neutralization capacity of the sera towards a divergent lyssavirus from phylogroup I, IRKV, and from phylogroup II, Mokola virus. Our findings revealed that RABVG-ME1-immunized mice produced IRK VNAs similar to the RABV group with no significant differences, indicating cross-reactivity within the same phylogroup. As expected, RABV∆G-MOKVG immune serum failed to neutralize IRKV (Figure 5B). Importantly, RABV-cAS1-vaccinated mice induced significantly higher MOKV-neutralizing antibodies compared to the RABV-vaccinated mice, indicating that antigenic site I from MOKVG can elicit MOKV VNAs (Figure 5C). However, MOKV neutralization was significantly higher (around 3-fold) upon vaccination with RABV∆G-MOKVG, suggesting that apart from antigenic site I, additional antigenic sites in MOKVG play a role in virus neutralization.

In summary, we found that the chimeric G antigen cAS1 acquired the ability to induce neutralizing antibodies to the phylogroup II lyssavirus MOKV while maintaining neutralization capacity for the phylogroup I lyssaviruses RABV and IRKV.

### 3.6. RABV-cAS1 Vaccine Efficacy against Lyssavirus Challenge

**Figure 6:**
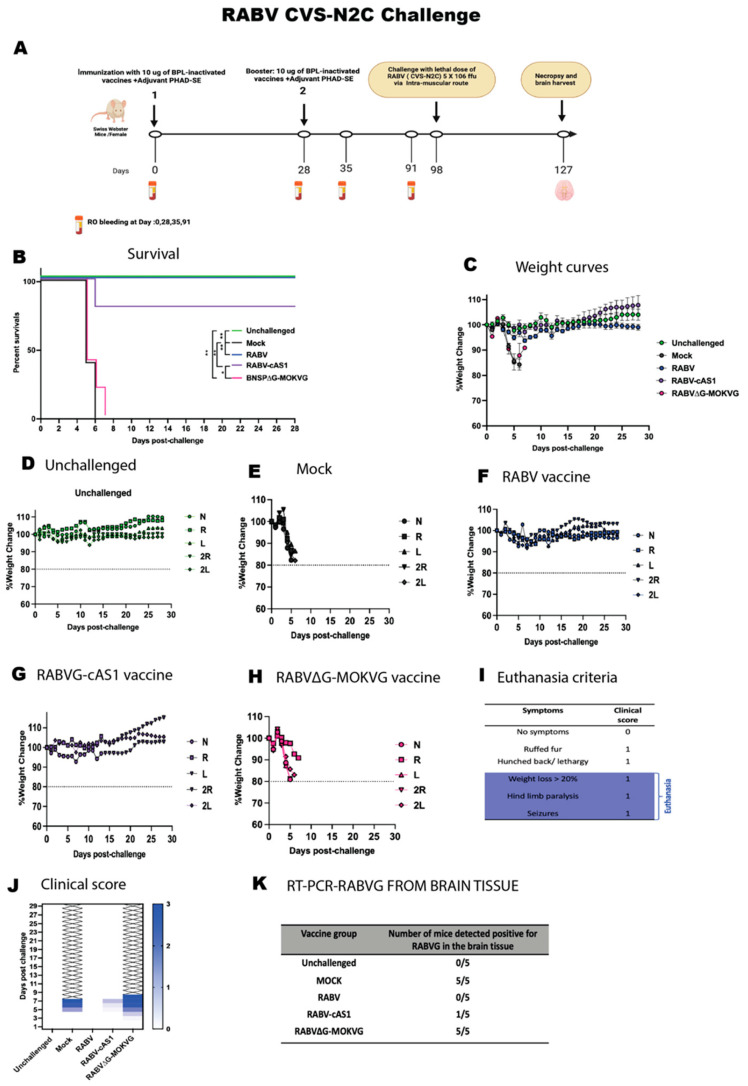
Challenge study to determine protection efficacy of RABV-cAS1 vaccine against RABV CVS-N2C. (**A**) Experimental timeline. Groups of 5 female mice were immunized with 10 µg/dose of BPL-inactivated vaccines adjuvanted with 5 µg of PHAD in 2% SE per dose. Day 98 indicates when mice were challenged with 5 × 10^6^ ffu of RABV CVS-N2C via intramuscular route. D127 and brain mark conclusion of study when any surviving mice were sacrificed. Created with Biorender.com (accessed 2 May 2024). (**B**) Kaplan–Meyer survival curves. Log-rank Mantel–Cox test was used to determine significance of survival of each group (** *p*  <  0.0021, * p  < 0.0332). (**C**) Group average weight change over time. Error bars represent standard deviation. (**D**) weight change over time for the unchallenged group. (**E**–**H**) indicate weight change over time per group; dotted line indicates 20% weight loss, N: mouse 1, R: mouse 2, L: mouse 3, 2R: mouse 4, 2L: mouse 5. (**I**) Euthanasia criteria and clinical scoring. (**J**) Daily cumulative clinical score per group. (**K**) RT-PCR-RABVG from brain tissues of each challenged group at end of study.

As neutralization is a good indication of protection from lyssavirus infection, we tested the effectiveness of the RABV-cAS1 vaccine in protection against lyssavirus phylogroup I and II challenge. Vaccinated mice of RABV-cAS1, RABV, RABV∆G-MOKVG, and mock-vaccinated mice were divided into three subgroups and challenged on day 91 with three different lyssaviruses: RABV, IRKV, and MOKV (see schedule and groups in Figure 4B). A lethal dose of 5.5 10^6^ FFU of RABV CVS-N2C and 10^5^ FFU of wt IRKV were injected i.m., while a lethal dose of 10^4^ FFU of wt MOKV was administered intranasally, as MOKV is not lethal to mice when injected i.m. (Supplementary Figure S2) [24]. When challenged with RABV (Figure 6), mock-vaccinated control mice lost weight and showed severe signs of disease, including paralysis, and were euthanized (Figure 6B,E), whereas RABV- and RABV-cAS1-vaccinated mice were both protected (100% and 80%, respectively, with no significant differences). The RABV∆G-MOKV-immunized mice did not survive the RABV challenge, as seen by weight loss and signs of disease, identical to the mock group (Figure 6B,H,J). The analysis of RABV RNA in mouse brains revealed that surviving mice immunized with RABV and RABV-cAS1 were protected from virus spread in the brain. While only one out of five mice vaccinated with RABVG-cAS1 failed the RABV challenge and was detected to be positive for RABVG RT-PCR as expected, RABV∆G-MOKV-vaccinated mice were all positive for RABV, confirming the virus spread in the brain, and the MOKV vaccine failed to protect against RABV (Figure 6K).

**Figure 7:**
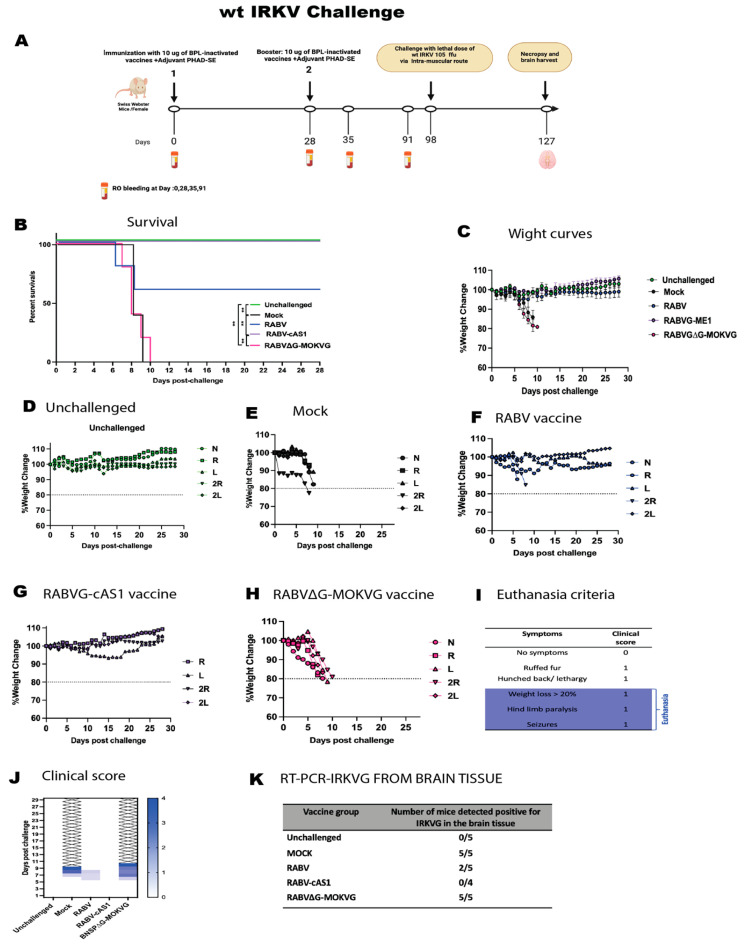
Challenge study to determine protection efficacy of RABV-cAS1 vaccine against wt IRKV. (**A**) Experimental timeline. Groups of 5 female mice were immunized with 10 µg/dose of BPL-inactivated vaccines adjuvanted with 5 µg of PHAD in 2% SE per dose. Day 98 indicates when mice were challenged with 10^5^ ffu of wt IRKV via intramuscular route. D127 and brain mark conclusion of study when any surviving mice were sacrificed. Created with Biorender.com (accessed 2 May 2024).. (**B**) Kaplan–Meyer survival curves. Log-rank Mantel–Cox test was used to determine significance of survival of each group (** *p*  <  0.0021). (**C**) Group average weight change over time. Error bars represent standard deviation. (**D**) weight change over time for the unchallenged group. (**E**–**H**) indicate weight change over time per group; dotted line indicates 20% weight loss, N: mouse 1, R: mouse 2, L: mouse 3, 2R: mouse 4, 2L: mouse 5. (**I**) Euthanasia criteria and clinical scoring. (**J**) Daily cumulative clinical score per group. (**K**) RT-PCR-RABVG from brain tissues of each challenged group at end of study. RABV-cAS1 group had only 4 mice.

When infected with wt IRKV (Figure 7), mock- and BNSP∆G-MOKVG-vaccinated mice lost weight and succumbed to infection within 9–10 days on average (Figure 7B,E,H). Each group was positive (five out of five) for IRKV G by RT-PCR confirming the IRKV spread in their brain (Figure 7K). Interestingly, the RABVG-cAS1 vaccination conferred 100% protection and completely prevented rabies symptoms, whereas the RABV vaccination provided 60% protection (Figure 7B,C). The RABV∆G-MOKV vaccine did not protect against IRKV or avoid symptoms of disease. (Figure 7H). The analysis of IRKV RNA in mouse brains confirmed that the RABV-cAS1 vaccine provided 100% protection against IRKV neuro-invasion, whereas two out of five RABV-vaccinated mice had detectable IRKV RNA (Figure 7K).

**Figure 8:**
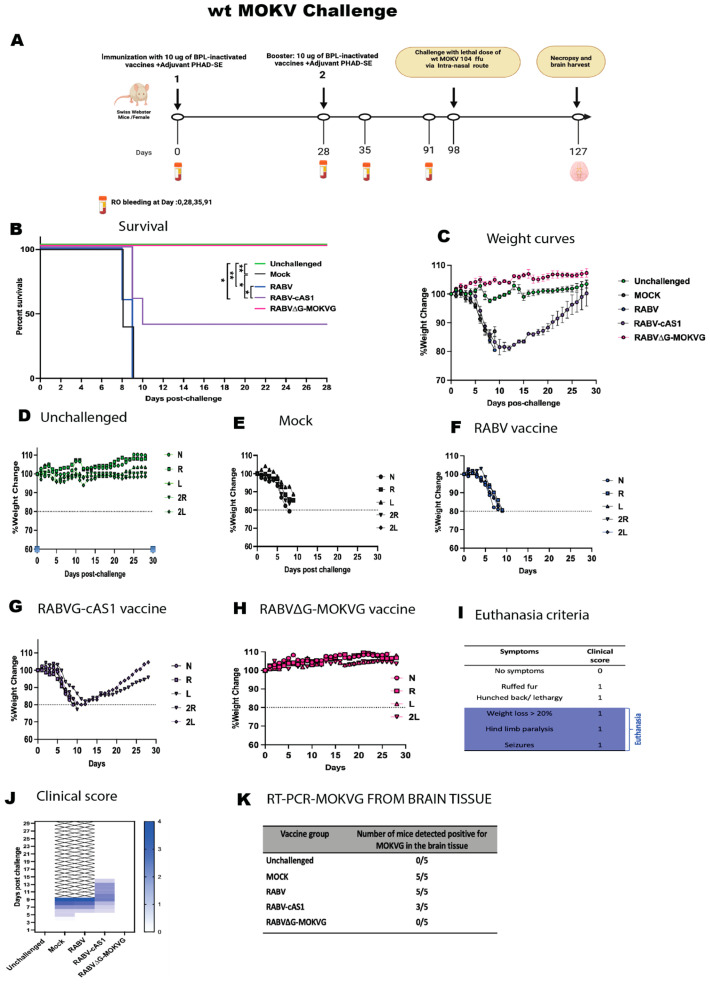
Challenge study to determine protection efficacy of RABV-cAS1 vaccine against wt MOKV. (**A**) Experimental timeline. Groups of 5 female mice were immunized with 10 µg/dose of BPL-inactivated vaccines adjuvanted with 5 µg of PHAD in 2% SE per dose. Day 98 indicates when mice were challenged with 10^5^ ffu of wt IRKV via intramuscular route. D127 and brain mark conclusion of study when any surviving mice were sacrificed. Created with Biorender.com. (**B**) Kaplan–Meyer survival curves. Log-rank Mantel–Cox test was used to determine significance of survival of each group (** *p*  <  0.0021, * p  < 0.0332). (**C**) Group average weight change over time. Error bars represent standard deviation. (**D**) weight change over time for the unchallenged group. (**E**–**H**) indicate weight change over time per group; dotted line indicates 20% weight loss, N: mouse 1, R: mouse 2, L: mouse 3, 2R: mouse 4, 2L: mouse 5. (**I**) Euthanasia criteria and clinical scoring. (**J**) Daily cumulative clinical score per group. (**K**) RT-PCR-RABVG from brain tissues of each challenged group at end of study.

Lastly, after the MOKV challenge (Figure 8), mock- and RABV-vaccinated mice rapidly lost weight and succumbed to infection within 8–9 days, while RABV∆G-MOKV vaccination conferred 100% protection (Figure 8B). Remarkably, RABV-cAS1-vaccinated mice had a 40% protection rate, with a significant difference from the RABV-vaccinated mice (Figure 8B). Two out of five mice were able to recover after weight loss, and no paralysis was observed in the RABV-cAS1-vaccinated mice (Figure 8C,G,J). The surviving mice from the RABVG-cAS1- or RABV∆G-MOKV-vaccinated groups had no detectable MOKV RNA in their brains. However, mock-vaccinated mice, RABV-vaccinated mice, and the non-survivor RABV-cAS1-vaccinated mice had detectable MOKV RNA in the brain.

In summary, our study showed that RABVG-cAS1 extended the protective capacity of rabies vaccination to IRKV, a distant phylogroup I virus; partially protected against MOKV, a phylogroup II virus; and, most importantly, maintained the protective capacity against RABV.

## 4. Discussion

The rapid expansion of structural biology and its associated tools, including computational biology, X-ray crystallography, and monoclonal antibody mapping, has solved many viruses’ glycoprotein structures, resulting in a revolution in vaccine development approaches [37]. In this paper, we proposed a next-generation vaccine against lyssavirus. Particular attention was paid to the AS1 of rabies and Mokola glycoproteins to develop the chimeric RABV-cAS1 vaccine design. RABV-cAS1 showed protection against divergent lyssaviruses of phylogroups I and II without compromising the immunogenicity and the protection capacity against RABV.

We demonstrated that, being a linear antigenic site [19,21], AS 1 (222 to 252) is a functional choice for substitution between two divergent lyssaviruses (RABV and MOKV) while maintaining a stable replicating recombinant virus. Previous studies have also sought to develop pan-lyssavirus vaccines using different approaches, such as utilizing a live vector to express lyssavirus glycoproteins, utilizing multiple vaccine constructs, and utilizing a recombinant rabies virus to express lyssavirus glycoproteins or one single chimeric glycoprotein [29,38]. The use of a live vector strategy utilizing recombinant rabies virus and recombinant vaccinia virus to express lyssavirus glycoproteins might be difficult to approve for human use for safety reasons [38,39]. The use of multiple vaccine constructs has been suggested, but this strategy would be costly, especially for low- and middle-income countries [40,41]. More recently, a recombinant RABV encoding two Gs (RABV G and MOKV G) resulted in lower virus growth, and the instability of the Gs was observed [29]. Therefore, the researcher created a single chimeric G composed of MOKV G and RABV G based on the pre- and post-fusion structures of a related rhabdovirus, vesicular stomatitis virus VSV-G [42]. While one of the RABVs that carried the chimeric G was stable (LyssaVax), the other showed an inefficient viral spread and low infectious viral titers [29].

Fisher et al. tested LyssaVax’s protective capability against two live viruses, rRABV and rMOKV, in an intranasal challenge. Although this route is artificial and does not resemble the natural rabies infection route, they demonstrated good protection against rRABV and rMOKV [29]. The same group investigated the serological pattern produced by LyssaVax against a panel of lyssaviruses from phylogroups I and II, but no phylogroup III was included. While LyssaVax stimulates VNA titers against phylogroup II viruses, it has been demonstrated that it loses some capacity in stimulating VNAs against non-RABV phylogroup I viruses, particularly IRKV and Duvenhage virus DUVV [29]. Fisher et al.’s research did show that the “domain” strategy they used to develop the vaccine scarified some loss of VNAs against divergent phylogroup I lyssaviruses.

By inserting one single antigenic side (MOKVG AS1) in the RABV-G vectored vaccine, we hypothesized an extension of immunogenicity against MOKV while preserving the immunogenicity against RABV. Our study showed high IgG titers against both MOKVG and RABVG. Meanwhile, some cross-reaction by ELISA between heterologous antigens was observed for sera from rabies-immunized mice, as shown in previous studies [29]; no VNAs were mediated by such antibodies.

We also hypothesized that the chimeric antigenic site (222–252) would provide broader protection against diverse lyssavirus challenges when compared to standard vaccines while still maintaining similar protection from rabies. RABV-cAS1 showed the potential of the AS1 of MOKV to elicit phylogroup II neutralizing antibodies while maintaining the VNA against phylogroup I. These outcomes align with other studies that have shown the specificity of each antigenic site in generating neutralization antibodies. Our results confirm the ability of an AS1-based vaccine approach to create a sustained immune response and specific neutralizing antibodies [43,44,45].

In our study, we demonstrated the potential of RABV-cAS1 to produce neutralizing antibodies against two divergent phylogroup I viruses, RABV and IRKV, with no significant difference. This appears to be a significant improvement in the chimeric lyssavirus vaccine via our novel approach, as the previous attempt published by Fisher et al. demonstrated the loss of VNA stimulation by LyssaVax against IRKV with and without adjuvant [26]. This might be related to the design utilized to create the chimeric G, where more antigenic portions of antigenic sites of RABVG were sacrificed. Of note, antigenic sites involved in the protection are not necessarily the same for all lyssaviruses. Cross-protection seen within phylogroup I by RABV vaccines has been described; however, the level of protection is undefined and might depend on high levels of G-specific antibodies [46,47,48]. Our findings are consistent with other investigations demonstrating that rabies vaccines confer the least protection against IRKV [48,49]. The most likely explanation for this limited protection is the antigenic variations within phylogroup I lyssaviruses, as IRKV is more closely related to European bat lyssavirus and Duvenhage virus than to rabies virus [1,50]. Comparison between RABV and IRKV showed that the antigenic sites I, II, and III were different, while the antigenic site IV was fully conserved [49,51].

While the cross-protection evaluation in this paper was limited to IRKV, this lyssavirus was reported in various research to display the highest pathogenicity in comparison to the other bat-associated lyssavirus, which explains why the existing RABV-based biologics give partial protection against the IRKV challenge [49,51]. Moreover, on the ancestral level, IRKV, RABV, ABLV, ARAV, BBLV, KHUV, DUVV, EBLV-1, and EBLV-2 segregated into one group, which suggests that serologic cross-reactivity should exist among these viruses [24,50]. Other investigations have divided phylogroup I into two main lineages: one that covers IRKV, EBLV-1, and DUVV and another lineage that includes the Palearctic (ARAV, BBLV, KHUV, and EBLV-2), Australian (ABLV), Oriental (GBLV), and American (RABV) lyssavirus species [52].

Interestingly, mice immunized with RABV-cAS1 were completely protected against wt IRKV, whereas RABV-immunized mice had limited protection. On one hand, AS1 contains a linear epitope with key residue LCGV, which is responsible for CR57 and 62071-3 mAbs binding. The alignment of IRKV AS1 and RABV AS1 revealed that IRKV loses V230. This amino acid change may alter the mAbs binding within this linear epitope, disrupting neutralization and limiting IRKV protection. Recent studies supported our findings, demonstrating that RABV vaccination is not completely effective against IRKV in animal models [49,51]. However, it is unclear how AS1 improved protection against IRKV when compared to RABV. The in silico analysis of RABV, IRKV, and MOKV AS1 showed that IRKV AS1 is more closely related to RABV AS1 with 64.5% identity, compared to 61.3% identity with MOKV AS1. However, two IRKV AS1 residues, R245 and D247, were shared with MOKV AS1 but not with RABV-cAS1 (Supplementary Figure S3). To confirm this, a future analysis focusing on these two residues may provide a better understanding of the gain of protection via MOKV AS1.

Our results highlight that while the MOKVG-immunized mice were fully protected, the RABV-cAS1 vaccine provided 40% protection, a significant improvement over the rabies vaccine, which does not protect against MOKV. These findings are in accordance with previous studies confirming that RABV vaccines fail to protect against phylogroup II lyssaviruses [24,25,53]. Although the protection against wtMOKV was partial, we think that it is not related to the vaccine schedule. It is important to highlight that the RABV-cASI vaccine was administrated as deactivated and adjuvanted with 5 µg of the TLR-4 agonist synthetic Monophosphoryl Lipid A (MPLA), 3D (6 A)-PHAD (PHAD), in a 2% squalene-in-oil emulsion (SE). Recent studies with rabies-based vectored vaccines showed that the administration of the inactivated vaccine adjuvanted with PHAD-SE using (0–28) clearly improves the immune responses elicited by the vaccine using [32,33,34].

Further, we found MOKVG in the postmortem brain tissue of mice that succumbed to disease but not in those that survived. It is unclear whether the vaccines were able to prevent the virus reaching the brain or clear it after entry. Post-challenge symptom data revealed that RABV-cAS1-immunized mice and challenged intranasally with wtMOKV developed ruffed fur, lethargy, and weight loss, but no paralysis was observed. While the controls were euthanized from days 9 to 10, the two survivor mice did not meet the euthanasia criteria and started to recover on day 11 upon the challenge. Based on the RT-PCR results from brain tissues shown in Figure 8K, the wt MOKV virus was found in the brain tissues of the three euthanized mice in the same group but not in these two survivors. Thus, we speculate that the virus reached the brain, and then the mice recovered, especially since the wt MOKV challenge was performed intranasally. This route was shown to be quick and assure the infection of the brain by penetration through both olfactory and trigeminal pathways [54,55,56]

Recovery from rabies post-infection is possible and has been reported in both human clinical infections and experimental infections in animals such as mice, dogs, ferrets, and rabbits [57,58,59]. Once the virus enters the CNS, it is protected from the humoral immune response unless an antibody can cross the BBB. Previous studies have found a correlation between recovery and both BBB permeability and antibody presence in cerebrospinal fluid [59,60,61]. According to published data, T cell activities can inhibit rabies virus replication [62,63], but the production of rabies virus-specific antibodies via B cell infiltration across the blood–brain barrier is critical for rabies virus elimination [17,61,64]. Under normal conditions, the BBB prevents contact between cells and factors in the bloodstream and those in the CNS. During the clearance of attenuated rabies virus from CNS tissues, the BBB opens to fluid-phase markers but not to larger molecules. This presumably allows immune cells in the neurovasculature to detect chemoattractants produced in the CNS and travel up the gradient across the BBB to infected tissues [61,64]. These findings are consistent with our data, reporting a recovery from rabies infection possibly after the virus has reached CNS tissues.

Our study proposes a novel approach towards the development of a pan-lyssavirus vaccine. However, this study had a few limitations. Since MOKV is not pathogenic via the intramuscular route, the MOKV challenge was performed via the intranasal route, an artificial route that increases the risk of severe disease due to its proximity to the CNS. Moreover, due to the limited sera, we are aware of the limited number of non-rabies lyssaviruses tested for cross-protection, and we think that threshold determination will aid further understanding. Protection studies against other phylogroup II lyssaviruses, such as the Lagos bat and Shimoni bat, may reveal greater protection using the same route of challenge to enable better data comparison.

Importantly, despite the novelty of this approach, complete protection against phylogroups I and II has not yet been achieved and must be extended to phylogroup III. Developing a pan-lyssavirus requires a new strategy that strikes a balance between producing a stable recombinant virus and a chimeric single glycoprotein with a wider range of antigens representative of the lyssavirus genus.

## 5. Conclusions

With a critical need for a pan-lyssavirus vaccine, our study proposed an innovative broad-spectrum lyssavirus vaccine strategy where the antigenic site 1 of RABVG was replaced with that of MOKVG. While maintaining the functionality of the chimeric RABV-G and its protective capacity against RABV, the chimeric vaccine was able to provide full protection against IRKV, a distant phylogroup I lyssavirus, and partial protection against MOKV, a distant phylogroup II lyssavirus. Our research study is the first step towards an efficient pan-lyssavirus vaccine protecting against diseases like rabies caused by multiple lyssaviruses, for which no vaccine or other countermeasures are currently available.

## Figures and Tables

**Table 1:** List of primers.

Primer	Sequence	Description
SB.38	5′-CTAACACCCCTCCCGTACGCCGCCACCATGGTCCCTCAGGCTCTGC-3′	Forward for IN-Fusion of RABVG-cAS1 into RABV vector with (BsiWi) restriction site
SB.39	5′-CATACAAAGGGCTCCCCGGTACTTGAG-3′	Reverse for IN-Fusion of RABVG-cAS1 RABV vector (Nhel) restriction site
RP951	5′-GGAGGTCGACTAAAGAGATCTCACATAC-3′	Sequencing of foreign gene in RABV vector between N and P
RP952	5′-TTCTTCAGCCATCTCAAGATCGGCCAGAC-3′	Sequencing of foreign gene in RABV vector between N and P
SB.28	5’- CCATCTAAGCTGCCCCAACA-3’	Forward for RT-PCR of wt Mokola virus glycoprotein (strain: Genbank: GQ500112.1)
SB.29	5′- AGATGCGGGACCCATTCATG-3′	Reverse for RT-PCR of wt Mokola virus glycoprotein (strain: Genbank: GQ500112.1)
SB.30	5′- TCGGGCTCCTCTTGTGTCTA-3′	Forward for RT-PCR of wt IRKV virus glycoprotein (strain: GenBank: EF614260.1)
SB.31	5′ CGCCTGCTGTCTTCCAACTA-3′	Reverse for RT-PCR of wt IRKV virus glycoprotein (strain: GenBank: EF614260.1)
SB.42	5′- CGTTATGGTGCCGTTAAATCGCTG-3′	Forward for RT-PCR Rabies CVS-N2C for glycoprotein (GenBank: HM535790.1)
SB.43	5′- TTGGACGGAGTTCAAGGAGGACTA-3′	Reverse for RT-PCR Rabies CVS-N2C for glycoprotein (GenBank: HM535790.1)

## Data Availability

Data are contained within the article and Supplementary Materials.

## Supplementary Materials

The following supporting information can be downloaded at https://www.mdpi.com/article/10.3390/v16071107/s1, Supplementary Figure S1: Expanded antigenic site 1 of RABV-cAS1. Supplementary Figure S2: A visualization of the structure of MOKVG and RABVG. Supplementary Figure S3: wt lyssavirus pathogenicity profiles. Supplementary Figure S4: Vaccines and MOKVG antigen characterization. Figure S5: Alignment of all lyssaviruses’ glycoproteins. Supplementary Figure S6: Challenge experiment summary. Supplementary Figure S7: Protection capacity of RABV-cAS1 against RABV, wtIRKV and wtMOKV.

## References

[1] Genus: Lyssavirus|ICTV. https://ictv.global/report/chapter/rhabdoviridae/rhabdoviridae/lyssavirus.

[2] Fisher C.R., Streicker D.G., Schnell M.J. (2018). The Spread and Evolution of Rabies Virus: Conquering New Frontiers. Nat. Rev. Microbiol..

[3] Calvelage S., Tammiranta N., Nokireki T., Gadd T., Eggerbauer E., Zaeck L.M., Potratz M., Wylezich C., Höper D., Müller T. (2021). Genetic and Antigenetic Characterization of the Novel Kotalahti Bat Lyssavirus (KBLV). Viruses.

[4] Rabies. https://www.who.int/news-room/fact-sheets/detail/rabies.

[5] Animals and Rabies|Rabies|CDC. https://www.cdc.gov/rabies/animals/index.html.

[6] Hanna J.N., Carney I.K., Smith G.A., Tannenberg A.E., Deverill J.E., Botha J.A., Serafin I.L., Harrower B.J., Fitzpatrick P.F., Searle J.W. (2000). Australian Bat Lyssavirus Infection: A Second Human Case, with a Long Incubation Period. Med. J. Aust..

[7] Weir D.L., Annand E.J., Reid P.A., Broder C.C. (2014). Recent Observations on Australian Bat Lyssavirus Tropism and Viral Entry. Viruses.

[8] Paweska J.T., Blumberg L.H., Liebenberg C., Hewlett R.H., Grobbelaar A.A., Leman P.A., Croft J.E., Nel L.H., Nutt L., Swanepoel R. (2006). Fatal Human Infection with Rabies-Related Duvenhage Virus, South Africa. Emerg. Infect. Dis..

[9] Poleshchuk E.M., Tagakova D.N., Sidorov G.N., Orlova T.S., Gordeiko N.S., Kaisarov A.Z. (2023). Lethal cases of lyssavirus encephalitis in humans after contact with bats in the Russian Far East in 2019–2021. Vopr. Virusol..

[10] Regnault B., Evrard B., Plu I., Dacheux L., Troadec E., Cozette P., Chrétien D., Duchesne M., Vallat J.-M., Jamet A. (2022). First Case of Lethal Encephalitis in Western Europe Due to European Bat Lyssavirus Type 1. Clin. Infect. Dis..

[11] Markotter W., Randles J., Rupprecht C.E., Sabeta C.T., Taylor P.J., Wandeler A.I., Nel L.H. (2006). Lagos Bat Virus, South Africa. Emerg. Infect. Dis..

[12] Coertse J., Markotter W., le Roux K., Stewart D., Sabeta C.T., Nel L.H. (2017). New Isolations of the Rabies-Related Mokola Virus from South Africa. BMC Vet. Res..

[13] Nel L., Jacobs J., Jaftha J., von Teichman B., Bingham J. (2000). New Cases of Mokola Virus Infection in South Africa: A Genotypic Comparison of Southern African Virus Isolates. Virus Genes.

[14] Ugolini G., Hemachudha T. (2018). Rabies: Changing Prophylaxis and New Insights in Pathophysiology. Curr. Opin. Infect. Dis..

[15] Warrell M.J. (2012). Current Rabies Vaccines and Prophylaxis Schedules: Preventing Rabies before and after Exposure. Travel. Med. Infect. Dis..

[16] O’Brien K.L., Nolan T. (2019). The WHO Position on Rabies Immunization—2018 Updates. Vaccine.

[17] Fooks A.R., Cliquet F., Finke S., Freuling C., Hemachudha T., Mani R.S., Müller T., Nadin-Davis S., Picard-Meyer E., Wilde H. (2017). Rabies. Nat. Rev. Dis. Primers.

[18] Yang F., Lin S., Ye F., Yang J., Qi J., Chen Z., Lin X., Wang J., Yue D., Cheng Y. (2020). Structural Analysis of Rabies Virus Glycoprotein Reveals pH-Dependent Conformational Changes and Interactions with a Neutralizing Antibody. Cell Host Microbe.

[19] Callaway H.M., Zyla D., Larrous F., de Melo G.D., Hastie K.M., Avalos R.D., Agarwal A., Corti D., Bourhy H., Saphire E.O. (2022). Structure of the Rabies Virus Glycoprotein Trimer Bound to a Prefusion-Specific Neutralizing Antibody. Sci. Adv..

[20] Shi C., Sun P., Yang P., Liu L., Tian L., Liu W., Wang M., Zheng X., Zheng W. (2022). Research Progress on Neutralizing Epitopes and Antibodies for the Rabies Virus. Infect. Med..

[21] Novel Rabies Virus-Neutralizing Epitope Recognized by Human Monoclonal Antibody: Fine Mapping and Escape Mutant Analysis. https://journals.asm.org/doi/epdf/10.1128/jvi.79.8.4672-4678.2005?src=getftr.

[22] Hellert J., Buchrieser J., Larrous F., Minola A., de Melo G.D., Soriaga L., England P., Haouz A., Telenti A., Schwartz O. (2020). Structure of the Prefusion-Locking Broadly Neutralizing Antibody RVC20 Bound to the Rabies Virus Glycoprotein. Nat. Commun..

[23] Malerczyk C., Freuling C., Gniel D., Giesen A., Selhorst T., Müller T. (2014). Cross-Neutralization of Antibodies Induced by Vaccination with Purified Chick Embryo Cell Vaccine (PCECV) against Different Lyssavirus Species. Hum. Vaccin. Immunother..

[24] Badrane H., Bahloul C., Perrin P., Tordo N. (2001). Evidence of Two Lyssavirus Phylogroups with Distinct Pathogenicity and Immunogenicity. J. Virol..

[25] Banyard A.C., Selden D., Wu G., Thorne L., Jennings D., Marston D., Finke S., Freuling C.M., Müller T., Echevarría J.E. (2018). Isolation, Antigenicity and Immunogenicity of Lleida Bat Lyssavirus. J. Gen. Virol..

[26] Viljoen N., Weyer J., Coertse J., Markotter W. (2023). Evaluation of Taxonomic Characteristics of Matlo and Phala Bat Rabies-Related Lyssaviruses Identified in South Africa. Viruses.

[27] Belot L., Ouldali M., Roche S., Legrand P., Gaudin Y., Albertini A.A. (2020). Crystal Structure of Mokola Virus Glycoprotein in Its Post-Fusion Conformation. PLoS Pathog..

[28] McGettigan J.P., Pomerantz R.J., Siler C.A., McKenna P.M., Foley H.D., Dietzschold B., Schnell M.J. (2003). Second-Generation Rabies Virus-Based Vaccine Vectors Expressing Human Immunodeficiency Virus Type 1 Gag Have Greatly Reduced Pathogenicity but Are Highly Immunogenic. J. Virol..

[29] Fisher C.R., Lowe D.E., Smith T.G., Yang Y., Hutson C.L., Wirblich C., Cingolani G., Schnell M.J. (2020). Lyssavirus Vaccine with a Chimeric Glycoprotein Protects across Phylogroups. Cell Rep..

[30] Schnell M.J., Mebatsion T., Conzelmann K.K. (1994). Infectious Rabies Viruses from Cloned cDNA. EMBO J..

[31] Kurup D., Wirblich C., Ramage H., Schnell M.J. (2020). Rabies Virus-Based COVID-19 Vaccine CORAVAX^TM^ Induces High Levels of Neutralizing Antibodies against SARS-CoV-2. NPJ Vaccines.

[32] Rios S., Bhattachan B., Vavilikolanu K., Kitsou C., Pal U., Schnell M.J. (2024). The Development of a Rabies Virus-Vectored Vaccine against Borrelia Burgdorferi, Targeting BBI39. Vaccines.

[33] Scher G., Bente D.A., Mears M.C., Cajimat M.N.B., Schnell M.J. (2023). GP38 as a Vaccine Target for Crimean-Congo Hemorrhagic Fever Virus. NPJ Vaccines.

[34] Yankowski C., Kurup D., Wirblich C., Schnell M.J. (2023). Effects of Adjuvants in a Rabies-Vectored Ebola Virus Vaccine on Protection from Surrogate Challenge. NPJ Vaccines.

[35] Blaney J.E., Wirblich C., Papaneri A.B., Johnson R.F., Myers C.J., Juelich T.L., Holbrook M.R., Freiberg A.N., Bernbaum J.G., Jahrling P.B. (2011). Inactivated or Live-Attenuated Bivalent Vaccines That Confer Protection against Rabies and Ebola Viruses. J. Virol..

[36] (2010). Rabies Vaccines: WHO Position Paper—Recommendations. Vaccine.

[37] Kryshtafovych A., Schwede T., Topf M., Fidelis K., Moult J. (2019). Critical Assessment of Methods of Protein Structure Prediction (CASP)-Round XIII. Proteins.

[38] Kgaladi J., Faber M., Dietzschold B., Nel L.H., Markotter W. (2017). Pathogenicity and Immunogenicity of Recombinant Rabies Viruses Expressing the Lagos Bat Virus Matrix and Glycoprotein: Perspectives for a Pan-Lyssavirus Vaccine. Trop. Med. Infect. Dis..

[39] Vuta V., Picard-Meyer E., Robardet E., Barboi G., Motiu R., Barbuceanu F., Vlagioiu C., Cliquet F. (2016). Vaccine-Induced Rabies Case in a Cow (Bos Taurus): Molecular Characterisation of Vaccine Strain in Brain Tissue. Vaccine.

[40] Viruses|Free Full-Text|Utilisation of Chimeric Lyssaviruses to Assess Vaccine Protection against Highly Divergent Lyssaviruses. https://www.mdpi.com/1999-4915/10/3/130.

[41] van de Burgwal L.H.M., Neevel A.M.G., Pittens C.C.M., Osterhaus A.D.M.E., Rupprecht C.E., Claassen E. (2017). Barriers to Innovation in Human Rabies Prophylaxis and Treatment: A Causal Analysis of Insights from Key Opinion Leaders and Literature. Zoonoses Public Health.

[42] Roche S., Rey F.A., Gaudin Y., Bressanelli S. (2007). Structure of the Prefusion Form of the Vesicular Stomatitis Virus Glycoprotein G. Science.

[43] Niu Y., Liu Y., Yang L., Qu H., Zhao J., Hu R., Li J., Liu W. (2016). Immunogenicity of Multi-Epitope-Based Vaccine Candidates Administered with the Adjuvant Gp96 against Rabies. Virol. Sin..

[44] Abdulhameed Odhar H., Hashim A.F., Humadi S.S., Ahjel S.W. (2023). Design and Construction of Multi Epitope- Peptide Vaccine Candidate for Rabies Virus. Bioinformation.

[45] Ding Y., Gao Y., Chen R., Zhang Z., Li Q., Jia T., Zhang T., Xu R., Shi W., Chen L. (2024). Development of a Novel Multi-Epitope Oral DNA Vaccine for Rabies Based on a Food-Borne Microbial Vector. Int. J. Biol. Macromol..

[46] Malerczyk C., Selhorst T., Tordo N., Moore S., Müller T. (2009). Antibodies Induced by Vaccination with Purified Chick Embryo Cell Culture Vaccine (PCECV) Cross-Neutralize Non-Classical Bat Lyssavirus Strains. Vaccine.

[47] Fooks A.R., Shipley R., Markotter W., Tordo N., Freuling C.M., Müller T., McElhinney L.M., Banyard A.C., Rupprecht C.E. (2021). Renewed Public Health Threat from Emerging Lyssaviruses. Viruses.

[48] Brookes S.M., Parsons G., Johnson N., McElhinney L.M., Fooks A.R. (2005). Rabies Human Diploid Cell Vaccine Elicits Cross-Neutralising and Cross-Protecting Immune Responses against European and Australian Bat Lyssaviruses. Vaccine.

[49] Liu Y., Chen Q., Zhang F., Zhang S., Li N., Lian H., Wang Y., Zhang J., Hu R. (2020). Evaluation of Rabies Biologics against Irkut Virus Isolated in China. J. Clin. Microbiol..

[50] Liu Y., Zhang S., Zhao J., Zhang F., Hu R. (2013). Isolation of Irkut Virus from a Murina Leucogaster Bat in China. PLoS Negl. Trop. Dis..

[51] Hanlon C.A., Kuzmin I.V., Blanton J.D., Weldon W.C., Manangan J.S., Rupprecht C.E. (2005). Efficacy of Rabies Biologics against New Lyssaviruses from Eurasia. Virus Res..

[52] Hayman D.T.S., Fooks A.R., Marston D.A., Garcia-R J.C. (2016). The Global Phylogeography of Lyssaviruses—Challenging the “Out of Africa” Hypothesis. PLoS Negl. Trop. Dis..

[53] Ogunkoya A.B., Beran G.W., Umoh J.U., Gomwalk N.E., Abdulkadir I.A. (1990). Serological Evidence of Infection of Dogs and Man in Nigeria by Lyssaviruses (Family Rhabdoviridae). Trans. R. Soc. Trop. Med. Hyg..

[54] Babic N., Mettenleiter T.C., Ugolini G., Flamand A., Coulon P. (1994). Propagation of Pseudorabies Virus in the Nervous System of the Mouse after Intranasal Inoculation. Virology.

[55] Lafay F., Coulon P., Astic L., Saucier D., Riche D., Holley A., Flamand A. (1991). Spread of the CVS Strain of Rabies Virus and of the Avirulent Mutant AvO1 along the Olfactory Pathways of the Mouse after Intranasal Inoculation. Virology.

[56] Griffin D.E. (2003). Immune Responses to RNA-Virus Infections of the CNS. Nat. Rev. Immunol..

[57] (2011). Recovery of a Patient from Clinical Rabies—California. https://www.cdc.gov/mmwr/preview/mmwrhtml/mm6104a1.htm.

[58] Manoj S., Mukherjee A., Johri S., Kumar K.V.S.H. (2016). Recovery from Rabies, a Universally Fatal Disease. Mil. Med. Res..

[59] Gold S., Donnelly C.A., Nouvellet P., Woodroffe R. (2020). Rabies Virus-Neutralising Antibodies in Healthy, Unvaccinated Individuals: What Do They Mean for Rabies Epidemiology?. PLoS Negl. Trop. Dis..

[60] Roy A., Phares T.W., Koprowski H., Hooper D.C. (2007). Failure to Open the Blood-Brain Barrier and Deliver Immune Effectors to Central Nervous System Tissues Leads to the Lethal Outcome of Silver-Haired Bat Rabies Virus Infection. J. Virol..

[61] Hooper D.C., Roy A., Barkhouse D.A., Li J., Kean R.B. (2011). Rabies Virus Clearance from the Central Nervous System. Adv. Virus Res..

[62] Mastraccio K.E., Huaman C., Coggins S.A., Clouse C., Rader M., Yan L., Mandal P., Hussain I., Ahmed A.E., Ho T. (2023). mAb Therapy Controls CNS-resident Lyssavirus Infection via a CD4 T Cell-dependent Mechanism. EMBO Mol. Med..

[63] Fabis M.J., Phares T.W., Kean R.B., Koprowski H., Hooper D.C. (2008). Blood-Brain Barrier Changes and Cell Invasion Differ between Therapeutic Immune Clearance of Neurotrophic Virus and CNS Autoimmunity. Proc. Natl. Acad. Sci. USA.

[64] Hooper D.C., Phares T.W., Fabis M.J., Roy A. (2009). The Production of Antibody by Invading B Cells Is Required for the Clearance of Rabies Virus from the Central Nervous System. PLoS Negl. Trop. Dis..

