# Supplementary material for: Toward the Development of a Pan-Lyssavirus Vaccine
Source: Viruses. 2024 Jul 10;16(7):1107. doi: 10.3390/v16071107 (PMC11281706; doi:10.3390/v16071107)
Supplement: Supplementary file 1 [file viruses-16-01107-s001.zip › viruses-3019138-supplementary.pdf]

# Supplementary figures

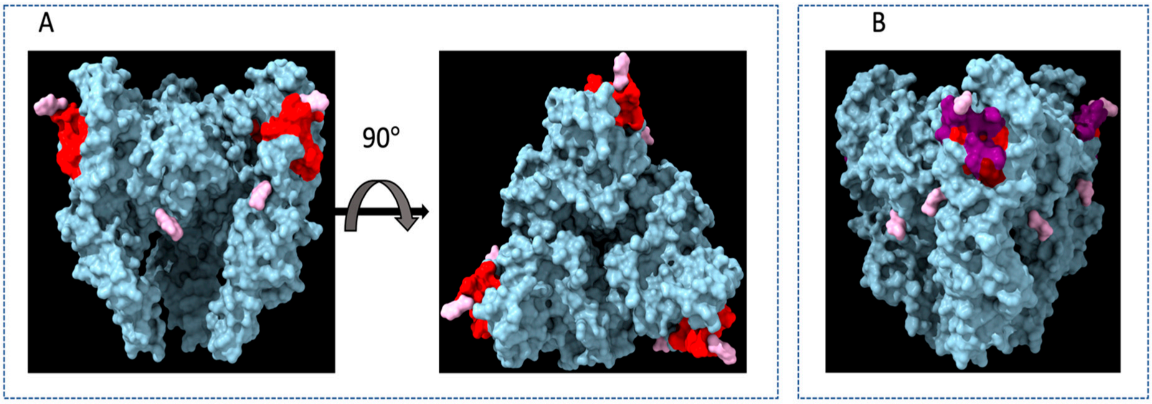


**Figure S1. Expanded antigenic site 1 of RABV-cAS1**

A: Prefusion trier for RABVG with the expanded antigenic site 1 highlighted in red (222-252): The amino acids 222-225, 227-229,232, 234-235,237-242,251-252 are from the rabies glycoprotein, while residues 226, 230-231, 233, and 236,243-250 are from the Mokola virus glycoprotein. B: Purple highlights the residues that have changed in the chimeric antigenic site. The N247 mutation induces a of the NAG glycosylation highlighted in pink. The figure was created with Chimera X (version 1.6rc202304140213, 2023-04-14).

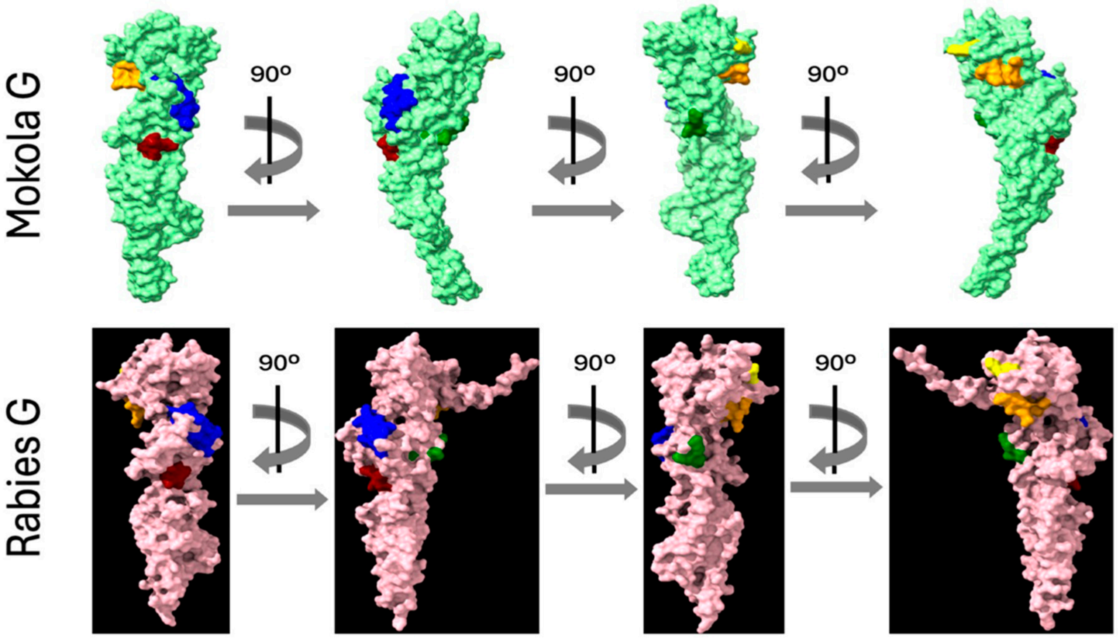


**Figure S2:** Structure of the RABVG and MOKVG showing the different antigenic sites as previously described [15]. The antigenic site 1: 226-231 is highlighted in maroon, antigenic site 2: 34-42, 198-200 is highlighted in blue, antigenic site 3: 330-338 is highlighted in orange, antigenic site 4: 261-264 is highlighted in green and minor antigenic site a: 342-343 highlighted in yellow.

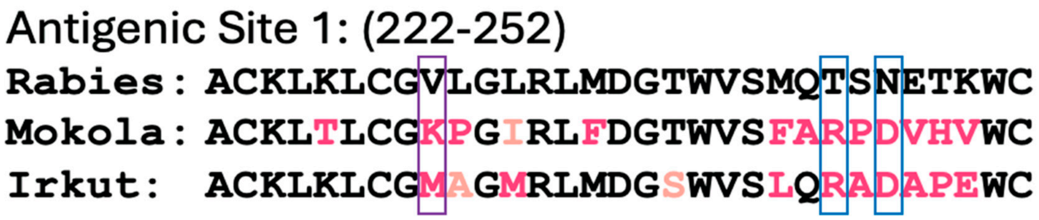


**Figure S3: AS1 of RABV, MOKV and IRKV Alignment**

Antigenic site 1 alignment. Dark pink indicates a non-conservative mutation relative to rabies, light pink indicates conservative. Purple square indicated a mutation in the linear epitope LCGV where CR57 and 62071-3 binds. Bleu squares indicates the shared residues R245 and D247 between MOKV and IRKV but not with RABV.

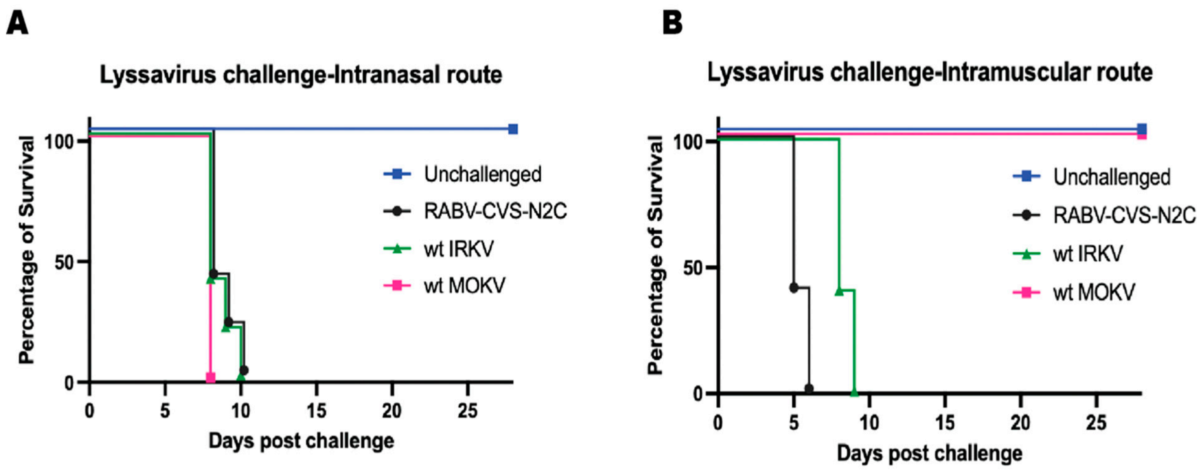


**Figure S 4. wt lyssaviruses pathogenicity profiles.**

A: Survival after lyssavirus intranasal (A) and Intramuscular challenge (B). Female Swiss webster mice were inoculated intranasally with $10^4$ ffu of lyssaviruses phylogroup I (Rabies Virus (CVS-N2C), Irkut virus), and phylogroup II (Mokola virus. B: Female Swiss Webster mice were inoculated intramuscularly with 6.5 X $10^5$ ffu of Rabies virus (CVS-N2C), $10^5$ ffu of Irkut virus, $10^5$ ffu of Mokola virus. Mice were monitored 28 days post challenge. Survival was analyzed using the log-rank Mantel-Cox test in comparison with the unchallenged mice group (A) **, P < 0.0021.

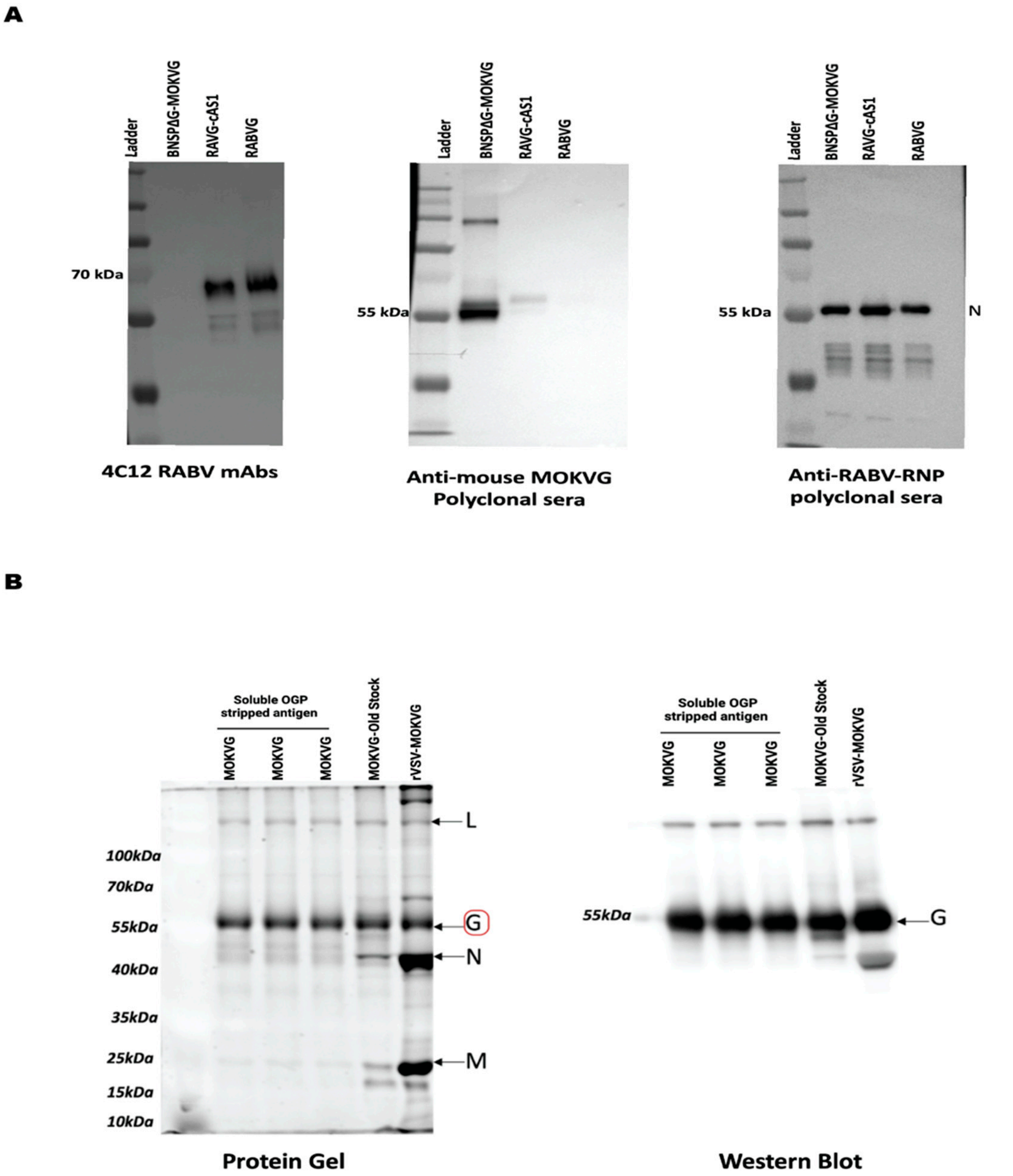


**Figure S5: Vaccines and MOKVG antigen characterization.**

A: Full gels of the vaccine's characterization probed against anti-RABVG-mAbs, anti-MOKVG polyclonal sera, and anti-Rabit-RABV-RNP. B SyproRuby stain and Western blot (against anti-MOKVG polyclonal sera) characterization for the MOKVG antigens striped from rVSV-MOKVG.

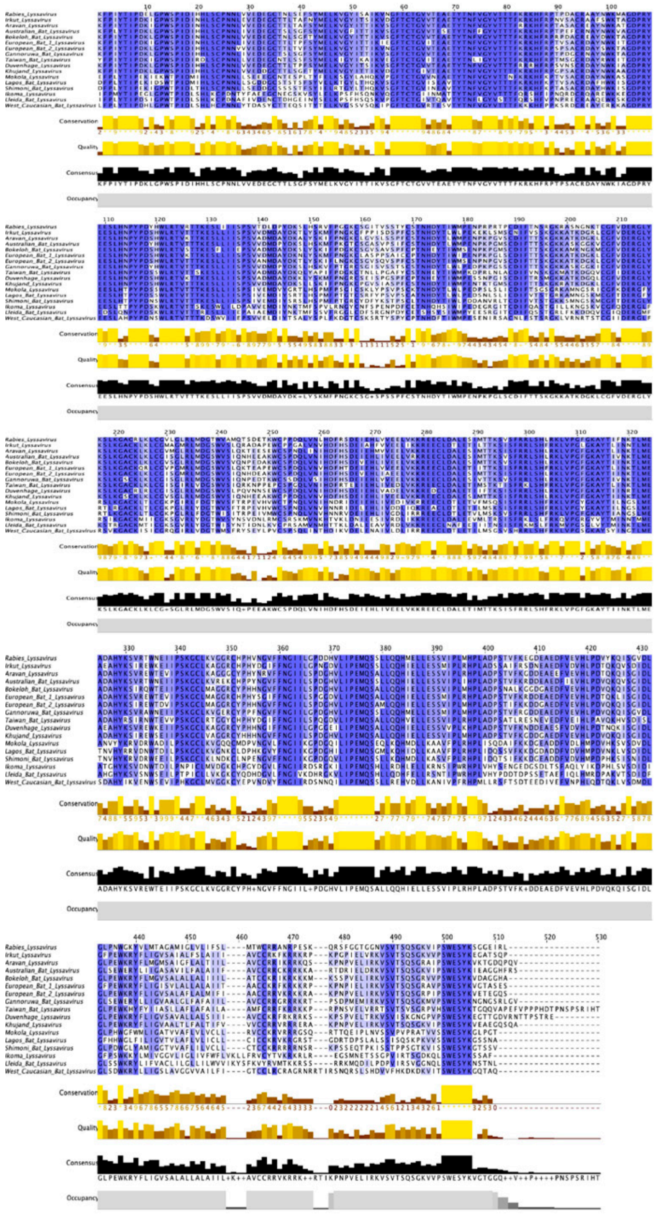

**Figure S6: Alignment of glycoproteins from different Lyssaviruses**: Signal peptide was excluded. Numbering on the top of the alignment denotes residue number omitting the signal peptide. Areas of relative conservation are denoted by blue shading. Ascension numbers to generate the alignment are as follows. Rabies: ADR03123.1, Irkut: AAR03480.1, Mokola: ACV86805.1, Lagos Bat: ADD84515.1, Gannoruwa Bat: APD77637.1, European Bat 1: ATY75015.1, European Bat 2: AUO29857.1, Bokeloh Bat: AWG43583.1, Australian Bat: QIN55368.1, Shimoni Bat: ADD84510.1, West Caucasian Bat: UGD08558.1, Lleida bat: YP_009325415.1, Aravan: AAP86775.1, Duvenhage: CAG9056782.1, Khujand: AAP86779.1, Ikoma: AFQ62097.1, Taiwan Bat: UUL86639.1

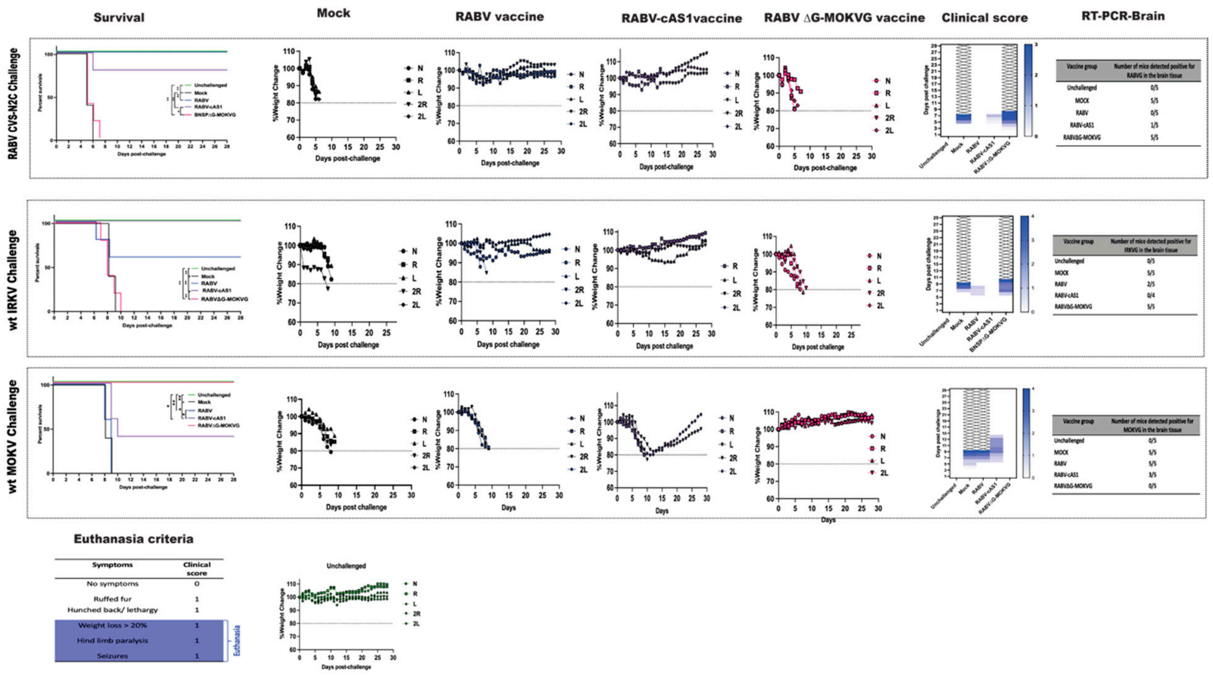


**Figure S7: Protection capacity of RABV-cAS1 against RABV, wt IRKV anf wtMOKV**

Summary of the challenge experiment. Groups of 5 female mice were immunized with 10 µg/dose of BPL inactivated vaccines adjuvanted with 5 µg of PHAD in 2% SE per dose. Day 98 indicates when mice were challenged with 5E6 ffu of RABV CVS-N2C via intramuscular route, 1E5 ffu of wt IRKV via intramuscular route and 1E4 ffu of wt MOKV via intranasal route.
